# Radial Endobronchial Ultrasound for the Diagnosis of Peripherally Located Pulmonary Lesions

**DOI:** 10.7759/cureus.87796

**Published:** 2025-07-12

**Authors:** Anish Mutum, Irom Ibungo Singh, Sabin Rai, Laishram Deepak Kumar

**Affiliations:** 1 Respiratory Medicine, Regional Institute of Medical Sciences (RIMS), Imphal, IND; 2 Pathology, Regional Institute of Medical Sciences (RIMS), Imphal, IND

**Keywords:** adenocarcinoma, diagnostic yield, minimally invasive, peripheral pulmonary lesions, pulmonary tuberculosis, radial ebus, transbronchial lung biopsy

## Abstract

Introduction

Peripheral pulmonary lesions (PPLs) pose a significant diagnostic challenge for the clinician as they are not visualized within the bronchial tree during flexible bronchoscopy. Radial endobronchial ultrasound (EBUS) is a minimally invasive bronchoscopic technique that expands the view of the bronchoscopist beyond the lumen of the airway, offering a promising diagnostic modality for PPLs.

Objectives

To assess the types of different peripheral pulmonary lesions diagnosed by radial endobronchial ultrasound-guided biopsy, and also to assess the agreement between the findings of radiological imaging and radial EBUS-guided biopsy in determining benign and malignant peripheral pulmonary lesions.

Methodology

This was a cross-sectional study performed on 40 patients aged ≥18 years with radiological evidence of PPLs who attended the respiratory medicine outpatient department (OPD) of the Regional Institute of Medical Sciences (RIMS), Imphal, Manipur, from April 2023 to March 2025.

Results

This study determined the diagnostic yield of radial EBUS to be 77.5%. Among the malignant lesions diagnosed, adenocarcinoma (15 patients, 37.5%) was the most common histopathological type followed by squamous cell carcinoma (eight patients, 20%) and small cell carcinoma (one patient, 2.5%) while benign lesions included pulmonary tuberculosis (five patients, 12.5%) and pulmonary aspergillosis (two patients, 5%). Substantial agreement (Kappa=0.674, p<0.001) was found between CT diagnosis and radial EBUS transbronchial lung biopsy (TBLB) findings in classifying lesions as benign or malignant.

Conclusion

In this study, radial EBUS-guided TBLB was found to be a safe and effective diagnostic modality for PPLs, offering high diagnostic yield and strong concordance with imaging findings, reinforcing its value in clinical decision-making.

## Introduction

Peripheral pulmonary lesions (PPLs) are defined as focal radiographic opacities that may be characterized as nodules (≤3 cm) or masses (>3 cm) [1]. PPLs are not visualized within the bronchial tree during flexible bronchoscopy. Differential diagnoses can include malignant and benign causes, such as infection and inflammation [2].

The diagnosis of PPL remains a significant challenge for the clinician [3]. Several options for the accurate diagnosis of PPL are available in major respiratory centers. It is usually necessary to choose between fluoroscopically guided bronchoscopy-transbronchial biopsy (TBB), CT-guided transthoracic aspiration, or radial EBUS-guided bronchoscopic procedures [4].

Fluoroscopically guided bronchoscopy or transbronchial biopsy shows variable success in the diagnosis of PPL, ranging from 20-50% depending on the visibility of the PPL, size of the lesion, and skill of the bronchoscopist [4]. Although the diagnostic yield from CT-guided procedures is significantly higher, they typically only generate cytological samples, which are insufficient for all the molecular and genetic studies required to diagnose lung cancer [4]. CT-guided procedures also have disadvantages of a high rate of pneumothorax (15-43%, with 4-18% requiring drainage) and a high risk of bleeding (1-27%) [4]. Radial EBUS-guided sampling techniques, on the other hand, have more advantages than both previously mentioned techniques.

Radial endobronchial ultrasound (EBUS) is a minimally invasive bronchoscopic technique that expands the view of the bronchoscopist beyond the lumen of the airway [4]. The radial probe EBUS technique has evolved since its implementation for peripheral nodules. Early accounts explained the use of a radial probe to detect lesions, after which the probe was taken out and biopsy tools were introduced, either with or without fluoroscopic help, through the bronchoscope into the same segmental bronchus [5]. The guide sheath technique followed this method, using a guide sheath as an extension of the flexible bronchoscope, acting as a conduit for biopsy instruments into the vicinity of a pulmonary nodule [6].

Based on variations in the echogenicity of the lesion and normal lung parenchyma, radial-probe EBUS can accurately identify pulmonary nodules or masses. According to studies, radial-probe EBUS increases the diagnosis rates of peripheral pulmonary nodules, especially those with a diameter of less than 2 cm [7]. Diagnostic yield of radial EBUS in the diagnosis of PPL is high, reducing the number of repeated bronchoscopies and diagnostic thoracotomies [4].

Radial EBUS-guided sampling procedures are relatively safer with a higher diagnostic yield, which can be performed on an outpatient basis. The radial EBUS procedure is a minimally invasive diagnostic modality that is not available in many parts of the country besides reputable high-end centers. The radial EBUS procedure is novel in our setting and available only in the Regional Institute of Medical Sciences (RIMS) in Manipur, North-Eastern India, in the Government Institute. Therefore, in this study, we are assessing the role of this diagnostic modality for the diagnosis of peripherally located pulmonary lesions in patients attending the respiratory medicine department of RIMS, Imphal, and to assess the various types of peripheral pulmonary lesions diagnosed by radial EBUS-guided biopsy.

## Materials and methods

This study was a hospital-based cross-sectional study conducted from April 2023 to March 2025. This study included all patients aged 18 years and above who attended the respiratory medicine outpatient department (OPD) at the Regional Institute of Medical Sciences (RIMS), Imphal, Manipur during the study period, had radiological evidence of peripheral lung opacities, and underwent radial EBUS for the diagnostic evaluation of peripheral pulmonary lesions. Patients who had endobronchial lesions visible on conventional bronchoscopy, patients with severe emphysema, pulmonary bullae adjacent to the lesion, cardiopulmonary insufficiency, bleeding disorders or coagulopathies, and those who did not provide informed consent were excluded from the study. 

The sample size was not calculated since all the patients meeting the inclusion criteria were included in the study. Consecutive sampling was used, and every subject meeting the inclusion criteria was selected. Forty patients meeting the inclusion criteria were included in this study. Ethical approval was obtained from the Research Ethics Board of RIMS, Imphal, before the beginning of the study, and valid written informed consent was also obtained from the patients.

Detailed histories of all the patients who participated in the study were recorded. They were subjected to a thorough, detailed clinical examination; blood investigations like liver function tests (LFT), kidney function tests (KFT), prothrombin time (PT), international normalized ratio (INR), and activated partial thromboplastin time (APTT) were done for all the patients. Mantoux test, sputum examination for acid-fast bacilli (AFB), and sputum cartridge-based nucleic acid amplification test (CBNAAT) were also done. Chest X-ray posteroanterior (PA) view and contrast-enhanced CT scan of the thorax were taken.

All the patients were subjected to examination of the airways by conventional bronchoscopy prior to the insertion of the radial EBUS probe. Radial EBUS-guided bronchoscopy was performed, and the targeted PPL was identified using endobronchial ultrasound imaging. Then, radial EBUS-guided transbronchial lung biopsy (TBLB) of the targeted PPL was performed, and the tissue samples obtained were sent for histopathological examination.

Data was analysed using SPSS V26 for Windows (IBM Inc., Armonk, New York). Descriptive statistics, like frequency and percentages, were used for all variables of interest. Kappa statistics were used for the agreement analysis.

Calculation of diagnostic yield

The diagnostic yield was defined as the proportion of procedures that resulted in a definitive diagnosis based on histopathological examination of the radial EBUS-guided TBLB samples. It was calculated using the following formula:

$\text{Diagnostic yield}\text{=}\frac{\text{Number of cases with definitive diagnosis}}{\text{Total number of procedures performed}}\times \text{100}\%$

Cases with inconclusive findings, such as those reported as only showing inflammatory changes or dysplasia without a definitive pathological diagnosis, were considered non-diagnostic for the purpose of this calculation.

Procedure details

Bronchoscopy was performed in a dedicated bronchoscopy suite by an interventional pulmonologist and the associates. The procedures were performed while the patients were under conscious sedation induced by midazolam and pentazocine in addition to the local anesthesia with lignocaine spray (15%) in the pharynx and with lignocaine 2% at the laryngeal inlet. The bronchoscope used was FUJIFILM EB 530 T (FUJIFILM Corporation, Japan), which had a 2.8 mm diameter instrument channel with a distal end diameter of 5.8 mm and an insertion tube diameter of 5.9 mm.

Ultra-miniature EBUS (UM-EBUS) was used to allow for improved assessment and sampling of peripheral pulmonary lesions. The ultra-miniature radial probe (P-series, PB-2020-M) with ultrasonic processor SP-900 and scanner RS-900 was used. It had a working length of 2150 mm, distal end diameter of 1.4 mm, and maximum proximal diameter of 1.9 mm with a frequency of 20 MHz +/- 15% (FUJIFILM Corporation, Japan).

When a lesion was reached with the probe, the usual normal lung ''snowstorm'' appearance was replaced by a focal ultrasound alteration that could be marked by fluoroscopy, a guide sheath (GS), or both fluoroscopy and GS. Traditionally, the GS could be left in place after localizing the lesion, allowing for guided lung biopsies using forceps, brush, or needle biopsy. In the present study, a guide sheath without fluoroscopy was used as fluoroscopic imaging was not available in our setting.

Biopsy targets were selected by reviewing available CT images. When possible, multiplanar reconstruction was used to create sagittal and coronal views, which were reviewed in conjunction with standard axial images. The presence or absence of a bronchus sign did not influence the determination to proceed with bronchoscopy. No additional image guidance software (virtual bronchoscopy or electromagnetic navigation software) was used to assist with procedure planning.

The bronchoscope was advanced into the bronchus of interest as far as possible under direct vision. Thereafter, the guide sheath (GS) covered radial probe EBUS was advanced through the working channel until resistance was met. After pulling back the probe slightly, ultrasound scanning was done to identify the target lesion. The probe was withdrawn, keeping the GS in position. The biopsy forceps were then introduced through the GS, and the lesion was sampled. The biopsied sample was fixed in 10% formalin and sent for histopathological examination. 

## Results

**Figure 1:**
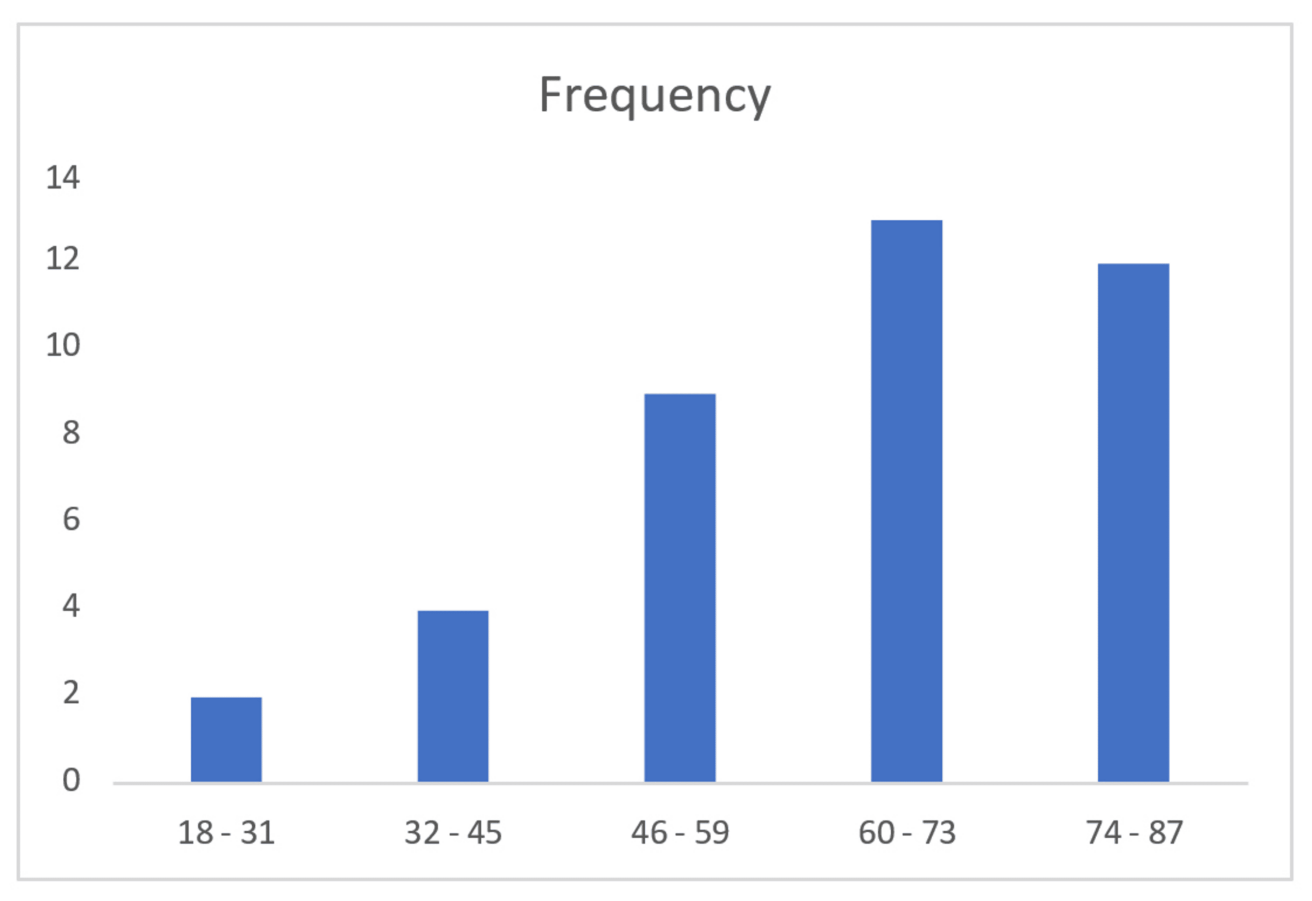
Age wise distribution of patients undergoing radial EBUS-guided biopsy EBUS - endobronchial ultrasound

Figure 1 shows that 13 patients were in the 60-73 age group, followed by 12 patients in the 74-87 age group. The lowest frequency was observed in the 18-31 age group, with only two patients. The mean age of the study population was 65.7 years, indicating that peripheral pulmonary lesions requiring diagnostic evaluation are more commonly observed in older individuals. 

**Figure 2:**
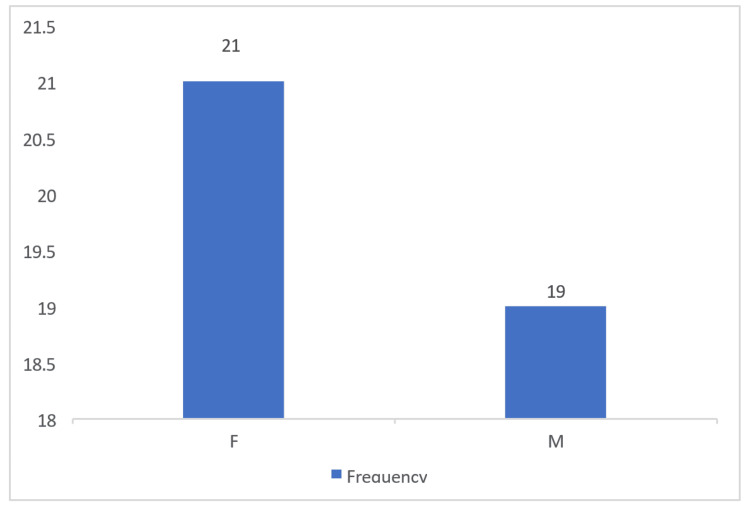
Sex distribution of patients undergoing radial EBUS-guided biopsy M - males; F - females; EBUS - endobronchial ultrasound

Figure 2 shows that out of the 40 patients with peripheral pulmonary lesions who underwent radial EBUS-guided biopsy, 21 (52.5%) were females, while 19 (47.5%) were males. This indicates a nearly equal distribution of cases between both genders, with a slightly higher prevalence among females. 

**Figure 3:**
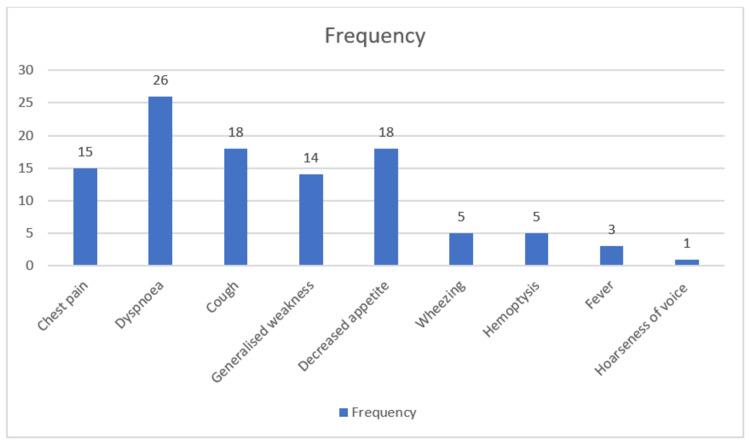
Prominent clinical symptoms at presentation

Figure 3 shows that among the 40 patients, dyspnoea (26 patients, 65%) was the most frequently reported symptom, followed by cough (18 patients, 45%) and decreased appetite (18 patients, 45%). Chest pain (15 patients, 38%) and generalized weakness (14 patients, 35%) were also common. Less frequently reported symptoms included wheezing (five patients, 13%), hemoptysis (five patients, 13%), fever (three patients, 8%), and hoarseness of voice (one patient, 3%). The predominance of respiratory symptoms aligns with the expected clinical presentation of pulmonary conditions.

**Figure 4:**
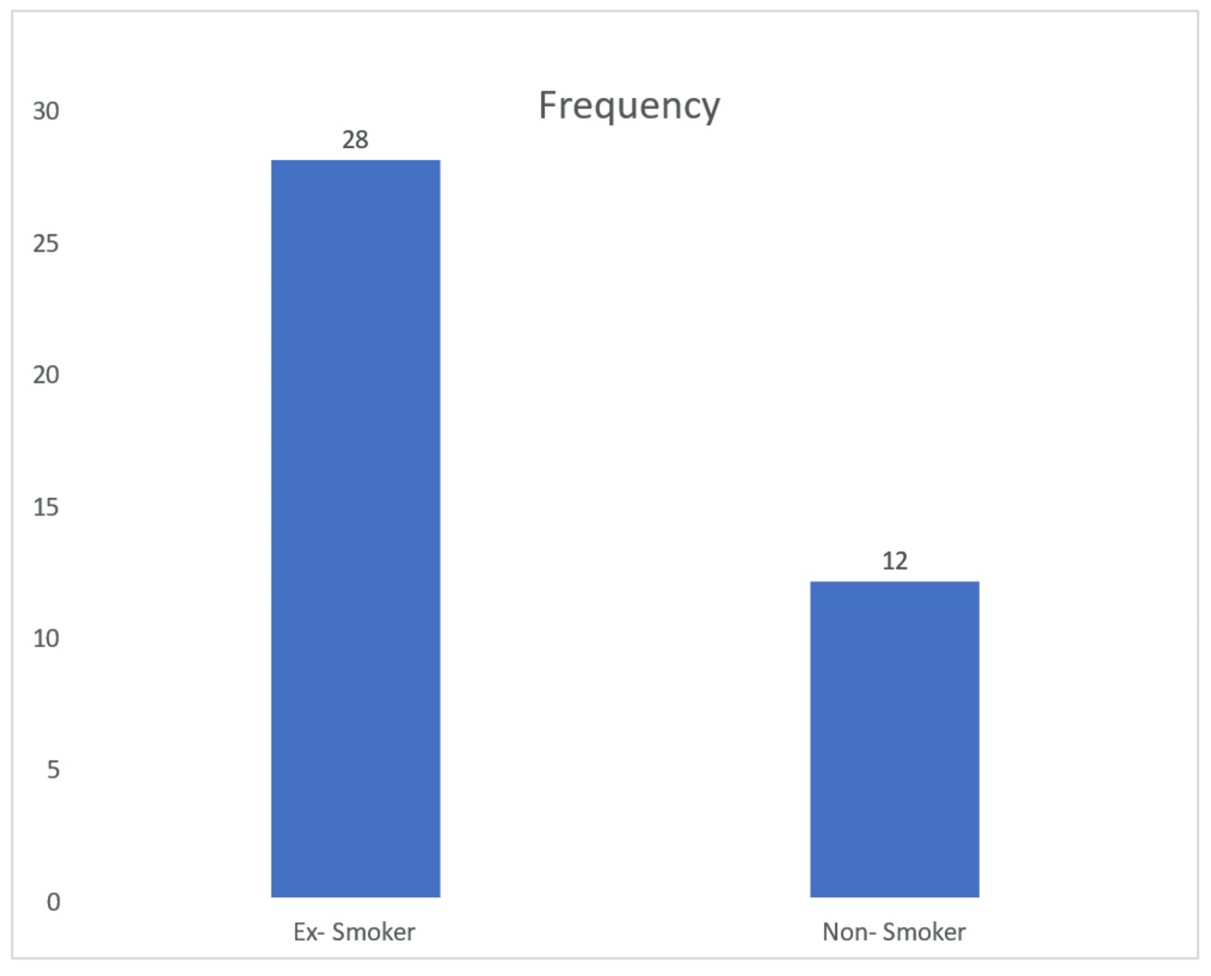
Smoking history of patients undergoing radial EBUS-guided biopsy EBUS - endobronchial ultrasound

Figure 4 shows that 28 patients (70%) were ex-smokers, while 12 patients (30%) were non-smokers. This shows that a significant proportion of patients with peripheral pulmonary lesions had a history of smoking, highlighting its potential role as a risk factor in the development of such lesions. 

**Figure 5:**
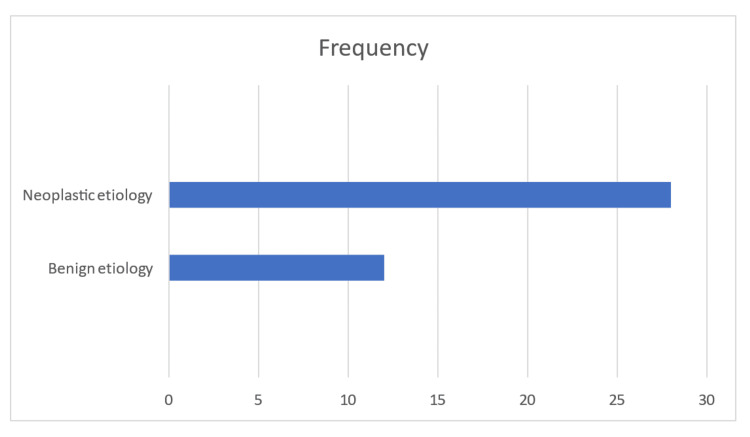
Computed tomography scan diagnosis

Figure 5 shows that based on CT scan findings, the majority of cases (28 patients, 70%) were diagnosed with neoplastic etiology, indicating a higher prevalence of malignancy in the study population. In contrast, 12 cases (30%) were classified as having benign etiology, suggesting the presence of non-malignant lung conditions. These findings highlight the importance of further diagnostic evaluation to confirm malignancy and guide appropriate management strategies. 

The findings from Table 1 indicate that adenocarcinoma was the most commonly diagnosed condition (15 patients, 37.5%), followed by squamous cell carcinoma (eight patients, 20%) and inflammatory changes (seven patients, 17.5%). Pulmonary tuberculosis was present in five cases (12.5%), while pulmonary aspergillosis and dysplasia each accounted for two patients (5%). Small cell carcinoma was the least frequent diagnosis (one patient, 2.5%). These results underscore the diagnostic efficacy of radial EBUS TBLB in identifying both malignant and benign lung lesions, thereby aiding in precise clinical decision making and treatment planning. 

**Table 1:** Radial EBUS TBLB findings and distribution of diagnoses EBUS - endobronchial ultrasound; TBLB - transbronchial lung biopsy

Radial EBUS TBLB findings	Frequency	Percent
Adenocarcinoma	15	37.5
Pulmonary aspergillosis	2	5
Dysplasia	2	5
Inflammatory changes	7	17.5
Small cell carcinoma	1	2.5
Squamous cell carcinoma	8	20
Pulmonary tuberculosis	5	12.5
Total	40	100

Calculation of diagnostic yield was as follows:

$\text{Total cases biopsied}\text{=}\text{40}$

$\text{Inconclusive results}\text{=}\text{9}$

$\text{Definitive diagnosis}\text{=}\text{40-9}\text{=}\text{31}$

$\text{Diagnostic yield}\text{=}\frac{\text{31}}{\text{40}}\times \text{100}\% \text{=}\text{77.5}\%$

Table 2 shows that among the 40 patients who underwent the procedure, 32 patients (80%) experienced no complications. However, bleeding (seven patients, 18%) was the most common complication, while pneumothorax occurred in one patient (3%). These findings indicate that while the procedure is generally safe, bleeding remains a notable concern.

**Table 2:** Frequency and percentage of procedural complications

Complications of the procedure	Frequency	Percent (out of 40 patients)
No complications	32	80%
Bleeding	7	18%
Pneumothorax	1	3%

Among the seven patients who experienced bleeding, all cases were classified as mild based on clinical assessment (self-limited bleeding or bleeding controlled without the need for additional procedures or transfusion). Management included topical instillation of cold saline through the bronchoscope’s working channel and observation. No patient required escalation of care or intervention beyond standard supportive measures.

Pneumothorax was identified in the chest radiograph PA view taken post-procedure, and it was managed conservatively with oxygen support.

Table 3 shows that the agreement between CT scan diagnosis and radial EBUS TBLB findings in classifying lesions as benign or malignant was substantial, with a kappa value of 0.674, indicating strong concordance. Among 12 cases identified as benign on CT scan, 11 were confirmed as benign, while one was found to be malignant. Conversely, out of 28 cases diagnosed as malignant on CT scan, 23 were confirmed as malignant, whereas five were found to be benign on radial EBUS TBLB. The statistically significant p-value (<0.001) highlights the reliability of CT scan in lesion classification while emphasizing the importance of radial EBUS TBLB for enhanced diagnostic accuracy. 

**Table 3:** Agreement between CT scan diagnosis and radial EBUS TBLB findings Number of valid cases = 40; measure of agreement (Cohen's Kappa) = 0.674; asymptotic standard error = 0.119; approximate T = 5.66 (approximate T indicates the test statistic used to assess the significance of the agreement measure, approximate T = Kappa/Standard error of Kappa); EBUS - endobronchial ultrasound; TBLB - transbronchial lung biopsy

CT scan diagnosis	Benign (radial EBUS TBLB finding)	Malignant (radial EBUS TBLB finding)	Total
Benign	11	1	12
Malignant	5	23	28
Total	16	24	40

**Figure 6:**
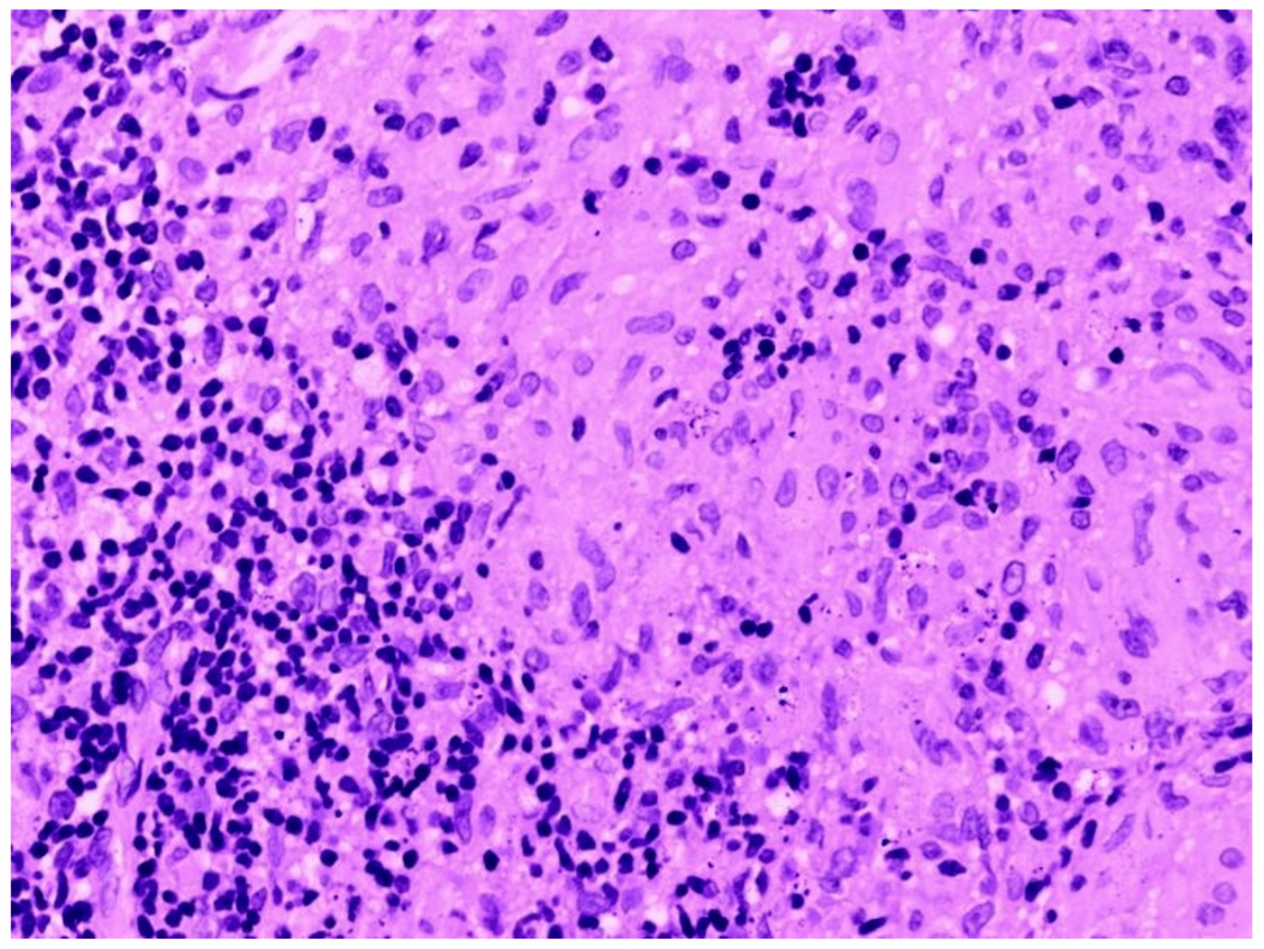
Histopathological examination of radial EBUS TBLB sample showing features of pulmonary tuberculosis EBUS - endobronchial ultrasound; TBLB - transbronchial lung biopsy

In Figure 6, histopathological examination (HPE) shows a coalescent epithelioid granuloma, which is suggestive of pulmonary tuberculosis.

**Figure 7:**
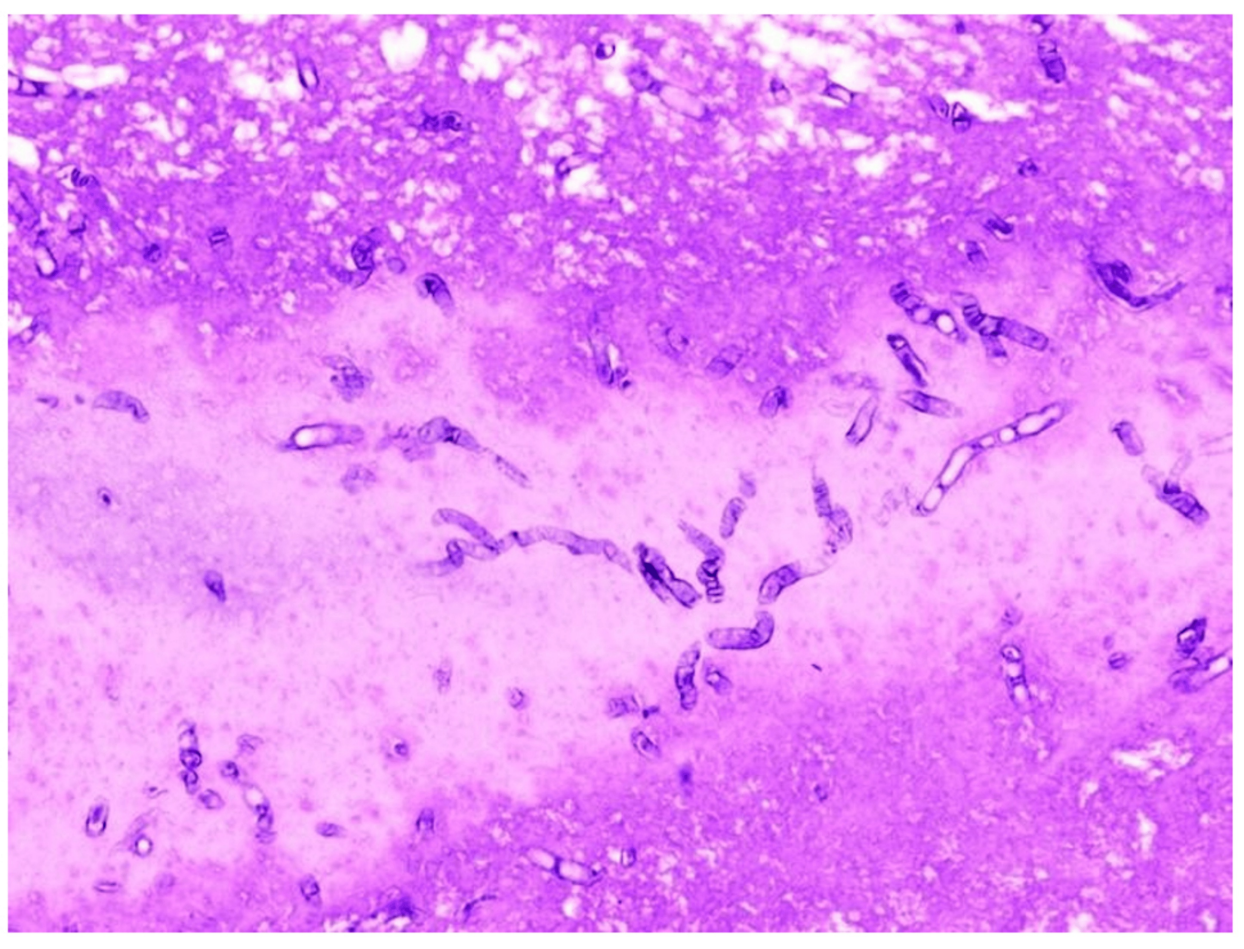
Histopathological examination of radial EBUS TBLB sample showing features of pulmonary aspergillosis EBUS - endobronchial ultrasound; TBLB - transbronchial lung biopsy

In Figure 7, HPE shows narrow septate hyphae branching at an acute angle, which is suggestive of pulmonary aspergillosis.

**Figure 8:**
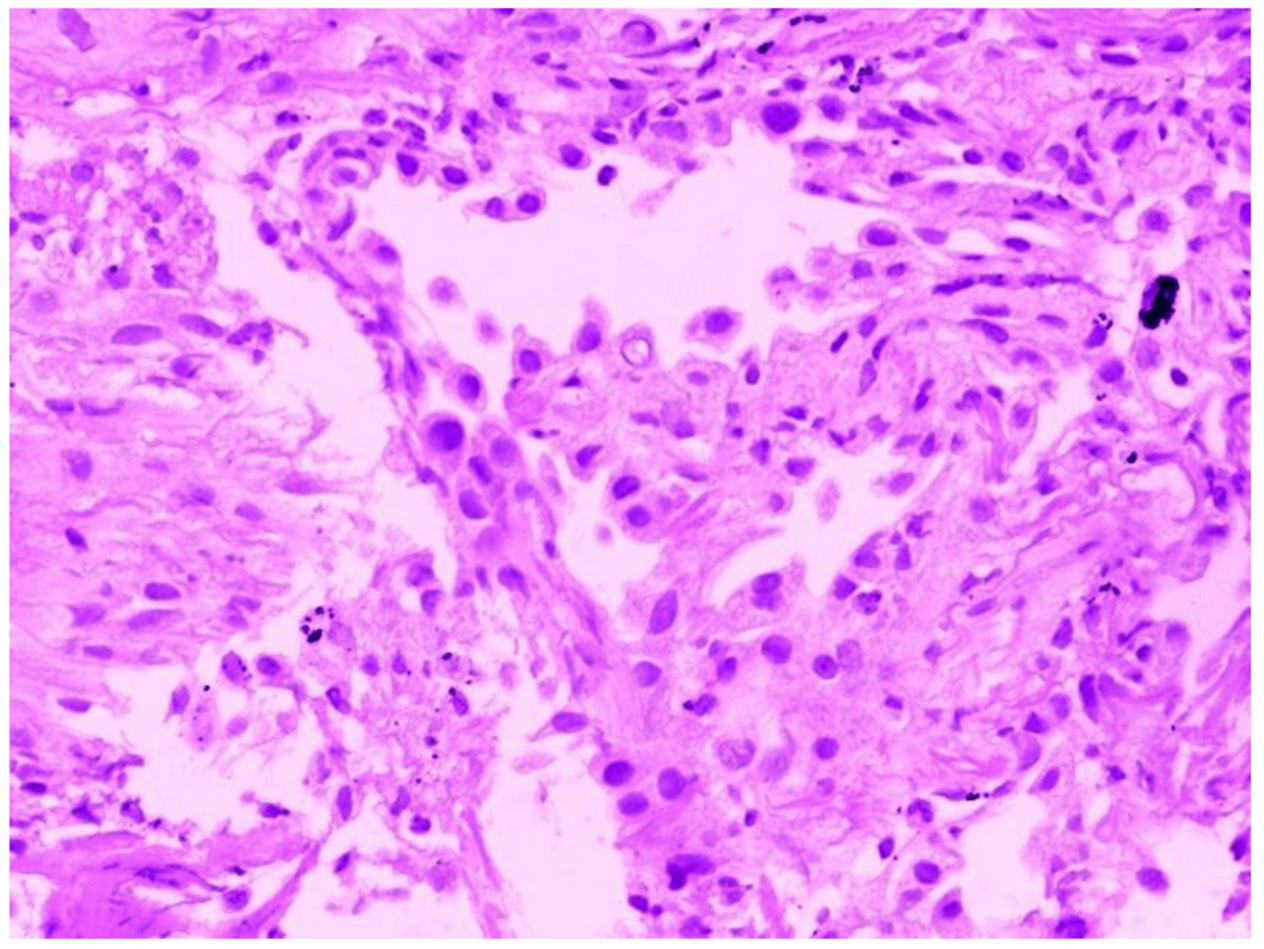
Histopathological examination of radial EBUS TBLB sample showing features of focal dysplasia EBUS - endobronchial ultrasound; TBLB - transbronchial lung biopsy

In Figure 8, HPE shows pulmonary alveoli lined by severely dysplastic epithelium with hyperchromatic nuclei and increased nuclear cytoplasmic ratio. Occasional cells show intranuclear cytoplasmic inclusion. These findings are consistent with dysplasia.

**Figure 9:**
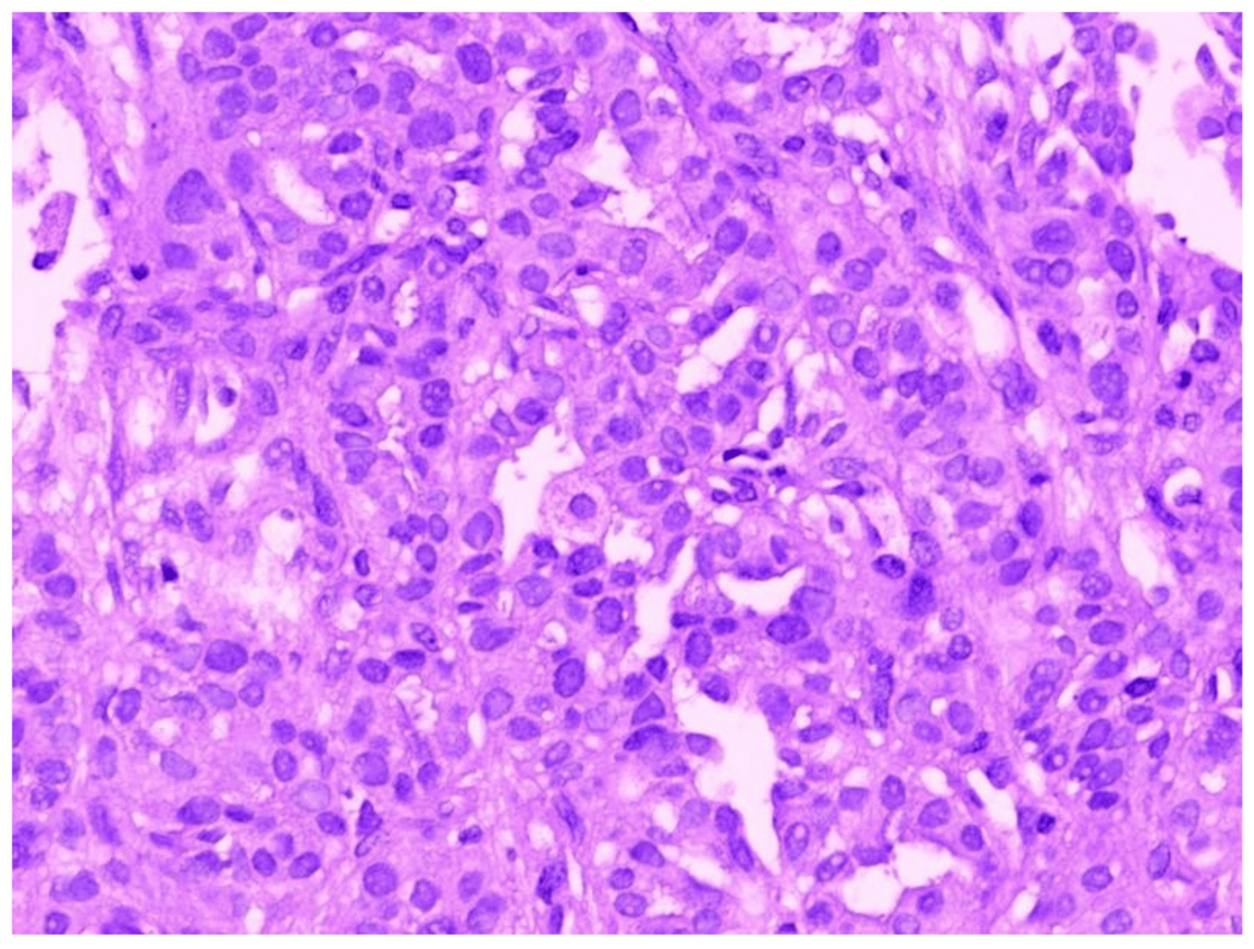
Histopathological examination of radial EBUS TBLB sample showing features of adenocarcinoma EBUS - endobronchial ultrasound; TBLB - transbronchial lung biopsy

In Figure 9, HPE shows tumour cells in solid sheets with areas of vague glandular formation, which is suggestive of adenocarcinoma.

**Figure 10:**
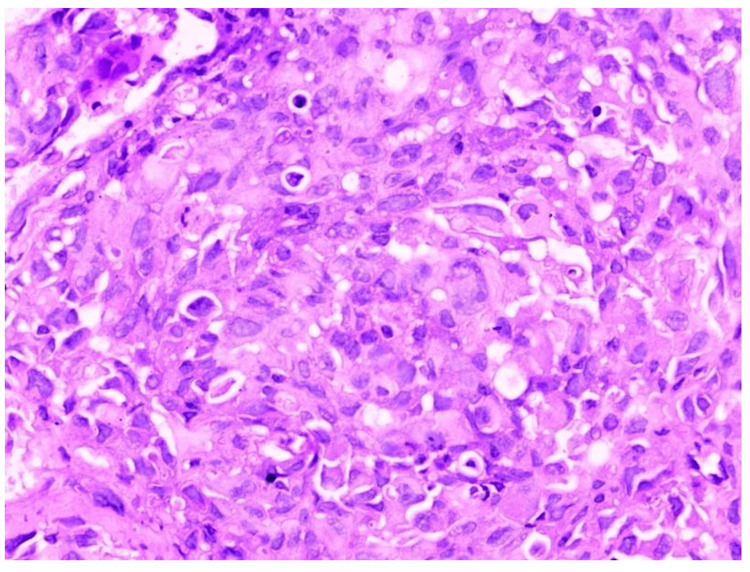
Histopathological examination (HPE) of radial EBUS TBLB sample showing features of squamous cell carcinoma

In Figure 10, HPE shows tumour cells in a diffuse sheet with intercellular bridges, which is suggestive of squamous cell carcinoma.

**Figure 11:**
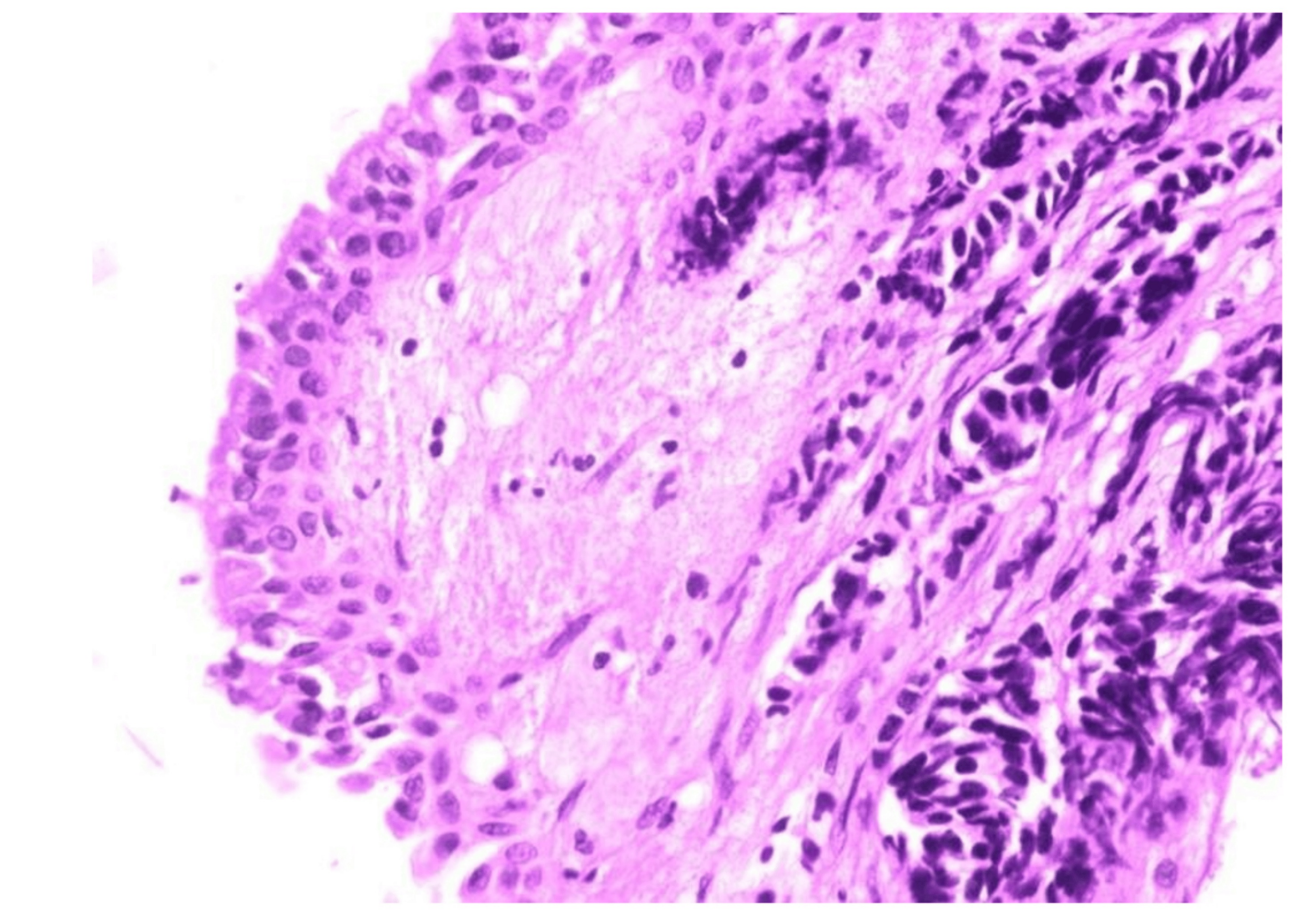
Histopathological examination of radial EBUS TBLB sample showing features of small cell carcinoma EBUS - endobronchial ultrasound; TBLB - transbronchial lung biopsy

In Figure 11, HPE shows bronchial tissue lined by dysplastic epithelium. Underlying mucosa shows hyperchromatic nuclei with scanty cytoplasm and nuclear moulding. These findings are suggestive of small cell carcinoma. 

## Discussion

The findings of this study underscore the diagnostic efficacy of radial probe endobronchial ultrasound (EBUS) in evaluating peripheral pulmonary lesions (PPLs). Our results reveal a substantial agreement (Kappa=0.674, p<0.001) between CT diagnosis and radial EBUS transbronchial lung biopsy (TBLB) findings in classifying lesions as benign or malignant. This high level of agreement suggests that radial EBUS serves as a reliable diagnostic modality, complementing CT scans in clinical decision making. The inclusion of the second objective in the study was intended to evaluate the reliability of radial EBUS as a confirmatory tool. While CT scan imaging remains an essential non-invasive method for assessing peripheral pulmonary lesions, it may not definitively distinguish benign from malignant etiologies, especially in regions with high prevalence of infectious diseases such as tuberculosis. This comparison helps validate the clinical utility of radial EBUS in confirming imaging findings and emphasizes its importance in guiding appropriate management strategies.

Previous studies, such as those conducted by Wang et al. [8] and Hong et al. [9], have consistently demonstrated radial EBUS's high diagnostic yield, which in our study was 77.5%, aligning with the findings of Tian et al. [10], who reported a similar diagnostic yield of 73.4% in their meta-analysis. Similarly, previous studies by Boonsarngsuk et al. [11], Lim et al. [12], and Xu et al. [13] had diagnostic yields of 79.9%, 71.6% and 70.3%, respectively. These findings reinforce the growing consensus that radial EBUS is an indispensable tool for pulmonologists managing PPLs.

The diagnostic profile of radial EBUS TBLB in this study demonstrates 77.42% of malignant lesions and 22.58% of benign lesions. Tay et al. [14] reported similar results, finding 69% malignant and 31% benign lesions. Xu et al. [13] also reported 67.3% of malignant lesions and 32.7% of benign lesions in their study.

In the present study, among the malignant lesions, adenocarcinoma (37.5%) was the most common malignancy identified, followed by squamous cell carcinoma (20%), while small cell carcinoma only accounted for 2.5%. In our study, benign lesions included pulmonary tuberculosis (12.5%) and pulmonary aspergillosis (5%). On the other hand, dysplasia and inflammatory changes accounted for 5% and 17.5% of cases, respectively, which were considered inconclusive findings as they did not aid in further definitive management of the patients.

These align with findings from Tian et al. [10] and Ito et al. [15], highlighting radial EBUS's accuracy in diagnosing lung malignancies. Additionally, our study detecting 12.5% of cases of pulmonary tuberculosis reaffirms research by Chang et al. [16] and Zhou et al. [17] that support radial EBUS's role in identifying infectious etiologies. Similar diagnostic distributions have been noted in studies by Park et al. [18], where adenocarcinoma remained the predominant malignancy detected via radial EBUS-guided biopsy. This ability to differentiate malignant from benign lesions is crucial in guiding timely and appropriate interventions. Given the high prevalence of tuberculosis in certain regions, these findings have significant clinical implications in distinguishing infections from malignancies.

Procedural complications were relatively rare, with 80% of patients experiencing no complications. However, bleeding occurred in 18% of cases and pneumothorax in 3%, emphasizing the need for careful procedural planning to mitigate risks. These complication rates align with prior research by Park et al. [18], who reported a 15-20% procedural complication rate in radial EBUS-guided biopsies.

Our study supports the expanding role of radial EBUS as a minimally invasive diagnostic approach where conventional bronchoscopy yields lower diagnostic accuracy. Studies by Hong et al. [9] and Park et al. [18] emphasize that radial EBUS, particularly when combined with a guide sheath, significantly improves diagnostic yield in ground-glass opacity lesions. Additionally, Brown et al. [19] and Xie et al. [20] highlight the enhanced efficacy of radial EBUS when paired with rapid on-site evaluation (ROSE) and cone beam CT, further improving diagnostic precision. These advancements indicate that radial EBUS is evolving into an indispensable diagnostic tool. Clinicians should consider incorporating these additional techniques to optimize procedural outcomes and reduce diagnostic uncertainties in peripheral lung lesions.

Recent meta-analyses by Tian et al. [10] and Chang et al. [16] reinforce the superior diagnostic yield of radial EBUS compared to conventional transbronchial biopsy techniques. Our study corroborates these findings and suggests that radial EBUS-TBLB should be integrated into routine clinical practice for evaluating PPLs.

While our study provides valuable insights into the diagnostic utility of radial EBUS, certain limitations must be acknowledged. First, the retrospective design may introduce selection bias, and the sample size, though robust, may not fully capture the heterogeneity of pulmonary lesions encountered in clinical practice. Second, while radial EBUS demonstrated a high diagnostic yield, false-negative results remain a concern, emphasizing the need for complementary diagnostic modalities such as cryobiopsy or advanced imaging techniques [17]. Third, operator experience and technical variability may impact diagnostic outcomes, necessitating standardized training protocols for EBUS operators. However, the strength of this study lies in its comprehensive analysis, substantial sample size, and direct comparison with CT findings, reinforcing the validity of our results. Further prospective studies should be conducted to address these limitations and optimize EBUS applications in real-world settings.

The results of this study reaffirm the clinical utility of radial EBUS in diagnosing peripheral pulmonary lesions, demonstrating substantial agreement with CT diagnosis and high diagnostic yield in malignant cases. These findings align with existing literature supporting the use of radial EBUS as a first-line diagnostic modality for PPLs. Future studies should explore the integration of adjunctive imaging techniques, such as radiomics and AI-driven diagnostics, to further enhance accuracy and improve patient outcomes. As technology continues to advance, the refinement of EBUS techniques and their combination with AI may provide even greater precision in lung lesion characterization. By leveraging these innovations, clinicians can improve early lung cancer detection, facilitating timely intervention, management, and better patient prognosis.

## Conclusions

This study highlights the significant diagnostic utility of radial probe endobronchial ultrasound (EBUS) in evaluating peripheral pulmonary lesions (PPLs). The findings demonstrate a substantial agreement between CT diagnosis and radial EBUS transbronchial lung biopsy (TBLB) in classifying lesions as benign or malignant. The high diagnostic yield, particularly for adenocarcinoma and squamous cell carcinoma, underscores the effectiveness of radial EBUS as a minimally invasive diagnostic tool. Additionally, its ability to detect infectious etiologies such as tuberculosis further establishes its broad clinical applicability. Our findings align with existing literature emphasizing the role of radial EBUS in improving diagnostic accuracy.

Integrating radial EBUS into routine clinical practice can significantly enhance the early detection and characterization of PPLs, especially in cases where conventional bronchoscopy has limited effectiveness and approaches. Comparative analyses confirm its superiority over traditional transbronchial biopsy techniques, making it a first-line approach for peripheral lung lesion evaluation. Emerging technologies such as optical coherence tomography and endoscopic navigation systems offer promising advancements that may further refine diagnostic precision in the future.

This study reaffirms the pivotal role of radial EBUS in diagnosing PPLs, demonstrating high accuracy and strong concordance with CT scan findings. It is a safe diagnostic intervention and holds great promise for advancing the field of interventional pulmonology.
